# Supplementary material for: Enhancement of polarizabilities of cylinders with cylinder-slab resonances
Source: Sci Rep. 2015 Feb 2;5:8189. doi: 10.1038/srep08189 (PMC4650704; doi:10.1038/srep08189)
Supplement: Supplementary Information — Enhancement of polarizabilities of cylinders with cylinder-slab resonances [file srep08189-s1.pdf]

Supplementary Information

Enhancement of polarizabilities of cylinders with cylinder-slab resonances

Meng Xiao[1], Xueqin Huang[1], H. Liu[2] and C. T. Chan[1*]

[1] Department of Physics and Institute for Advanced Study, the Hong Kong University of Science and Technology, Clear Water Bay, Hong Kong, China

[2] National Laboratory of Solid State Microstructures & Department of Physics, Nanjing University, Nanjing 210093, People's Republic of China

S-I. Asymptotic behavior of W_{yy}^{ref} in the limiting process of $z_d \to 0$ when $\omega \neq \omega_c$

$$W_{yy}^{ref} = \int_{-\infty}^{\infty} dk_p \frac{i}{4\pi k_z} \exp\left(2ik_z z_d\right) R\left(k_p\right) \quad \text{(S1)}$$

We note that $R\left(k_p\right)$ is small for $k_p < k_0$ when $\delta \to 0$, so the contribution of $k_p < k_0$ inside the integration in Eq. (S1) is negligible. For a large enough k_p, $R\left(k_p\right) \sim \frac{\varepsilon - 1}{\varepsilon + 1}$, $k_z \sim ik_p$, then

$$\begin{aligned}
W_{yy}^{ref} &= \int_{-\infty}^{\infty} dk_p \frac{i}{4\pi k_z} \exp\left(2ik_z z_d\right) R\left(k_p\right) \\
&\approx \int_{k_0}^{\infty} dk_p \frac{i}{2\pi k_z} \exp\left(2ik_z z_d\right) R\left(k_p\right) \\
&\approx \frac{\varepsilon_c - 1}{2\pi\left(\varepsilon_c + 1\right)} \int_{\kappa_0}^{\infty} dk_p \frac{1}{k_p} \exp\left(-2k_p z_d\right) \qquad \text{(S2)} \\
&= \frac{\varepsilon_c - 1}{2\pi\left(\varepsilon_c + 1\right)} \mathrm{Gamma}\left[0, 2\kappa_0 z_d\right] \\
&\sim \frac{1 - \varepsilon_c}{2\pi\left(\varepsilon_c + 1\right)} \mathrm{Log}\left[z_d/\mathrm{d}\right] \sim -\frac{1}{\pi\left(\varepsilon_c + 1\right)} \mathrm{Log}\left[\mathrm{d}/z_d\right]
\end{aligned}$$

where κ_0 represents a large enough k_p and it does not enter the final asymptotic behavior in the limiting process of $z_d \to 0$. In the last step, we use the condition that $\varepsilon_c \approx -1$ near ω_c.

To see whether our approximation works or not, we define two functions,

$$f\left(k_p\right) = \frac{i}{4\pi k_z} \exp\left(2ik_z z_d\right) R\left(k_p\right), \text{(S3)}$$

$$g\left(k_p\right) = \frac{\varepsilon_c - 1}{4\pi\left(\varepsilon_c + 1\right)} \frac{1}{k_p} \exp\left(-2k_p z_d\right). \text{(S4)}$$

$f\left(k_p\right)$ and $g\left(k_p\right)$ are the functions inside the integrations before and after the approximation in Eq. (S2). In the limiting process of $z_d \to 0$, the dominant part of W_{yy}^{ref} come from large k_p components where our approximation works well and $f\left(k_p\right) \approx g\left(k_p\right)$. In Fig. S1, we show the real part (a) and imaginary part (b) of $f\left(k_p\right)$ (black line) and $g\left(k_p\right)$ (red line) as a function of

k_p. In this figure, $\omega = 0.99645\omega_c$ and $z_d = 0.02d$. It is clear that for large enough k_z, $f(k_p) \approx g(k_p)$. Comparing Figs. S1 (a) and S1 (b), we know that, the imaginary part of the reflection Green function is orders of magnitude smaller than the real part.

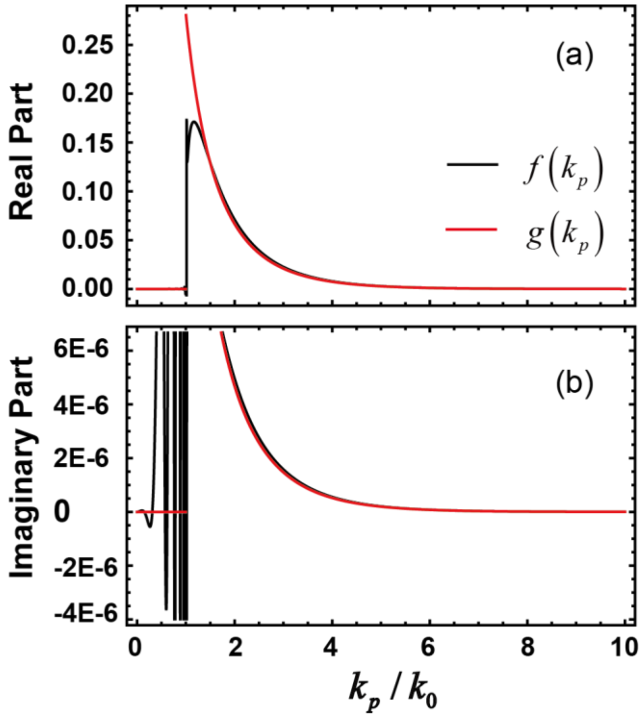

Fig. S1 (a) Real and (b) imaginary part of $f(k_p)$ and $g(k_p)$ as a function of k_p. $\omega = 0.99645\omega_c$ and $z_d = 0.02d$ in this figure. The parameters of the metamaterial slab are the same as those in the main text.

In Fig. S2 we show some numerical tests for the asymptotic behavior of the reflection Green function. The parameters of the metamaterial slab are the same as those in the main text. In Fig. S2(a), we keep $\omega = 0.99645\omega_c$ and vary z_d. It is clear that when z_d is small enough, $\mathrm{Re}\left[W_{yy}^{ref}\right]$ is proportional to $\mathrm{Log}\left[d/z_d\right]$; while when z_d is not small enough, $\mathrm{Re}\left[W_{yy}^{ref}\right]$ deviates from the straight line and becomes close to zero. For all the z_d under consideration in Fig. S2(a), $\mathrm{Re}\left[W_{yy}^{ref}\right] \gg \mathrm{Im}\left[W_{yy}^{ref}\right]$. In Fig. S2(b), we keep $z_d = 0.05d$ and vary the frequency.

W_{yy}^{ref} shows a resonance behavior around ω_c, and this resonance comes from the factor $\frac{-1}{(\varepsilon_c+1)}$.

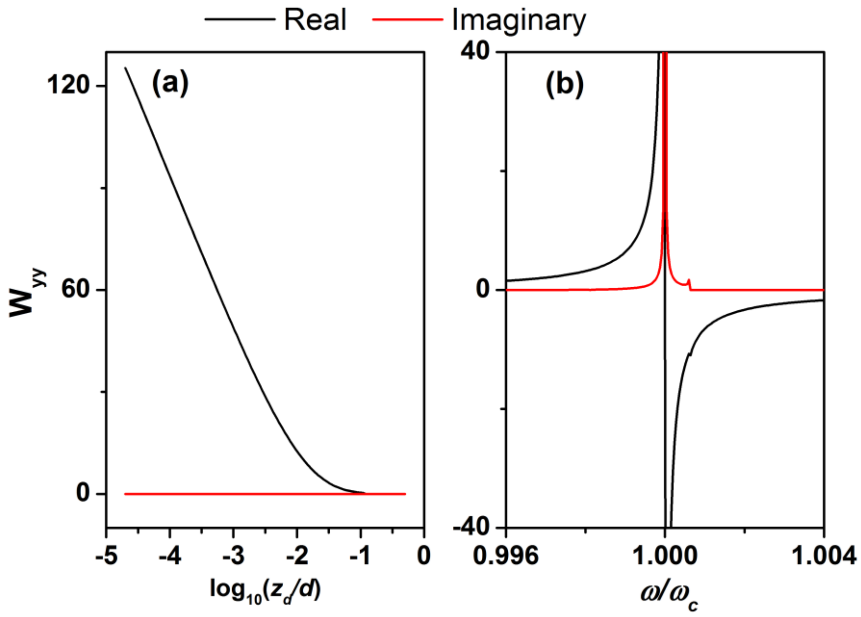

Fig. S2 (a) W_{yy}^{ref} as a function of z_d when $\omega = 0.99645\omega_c$. (b) W_{yy}^{ref} as a function of ω when $z_d = 0.05d$. In both (a) and (b), the black line and red line represent the real part and imaginary part of W_{yy}^{ref}, respectively. The parameters of the metamaterial slab are the same as those in the main text.

S-II. Force and Energy Barrier with Experimentally Achievable Parameters

In the main text, we showed that the force acting on the cylinder will be greatly enhanced by the cylinder-slab resonance mode. Here, we consider some concrete examples with experimentally achievable parameters. We assume that the working wavelength of the light source is around $\lambda_c = 2\mu\text{m}$. The absolute value is not important as Maxwell Equations are scale invariant. With the parameters considered in Fig. 4(a), we now have $d = 6\mu\text{m}$, $r_c = 30\text{nm}$, $\omega_c = 3\pi\times10^{14}[1/\text{s}]$ and $\varepsilon_c = 6$.

As a first demonstration, we calculate the force acting on the cylinder as a function of z_d, which is

the distance between the cylinder and the slab. The working frequency of incident light is set at $\omega = 0.998\omega_c$. Since the force is proportional to the power of the incident light field and the length of the cylinder, we show the force along z direction for unit power ($\mathrm{mW/\mu m^2}$) and unit length ($\mu\mathrm{m}$) of the small cylinder in Fig. S3. With increasing z_d, the force increases until reaching a maximum corresponding to the cylinder-slab resonance, then decreases to the value of force without the slab when the particle is far away. As a comparison, we also calculate the force without the slab, which is $2.88\times10^{-3}\mathrm{pN\,mW^{-1}}\mu\mathrm{m}$. This relative small value is due to the fact that the wave length is much larger than the radius of the cylinder.

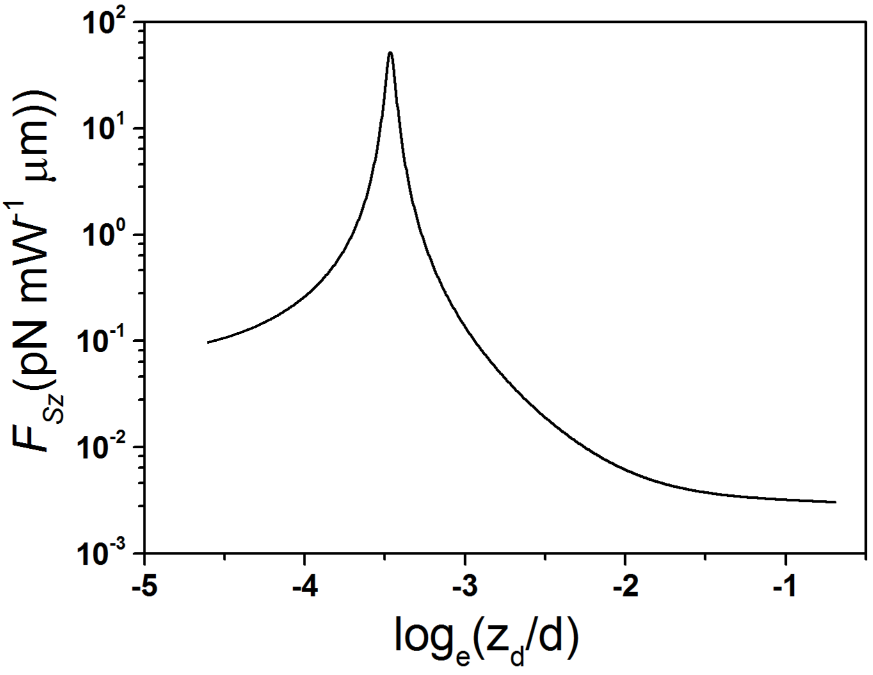

Fig.S3 Force acting on the cylinder as a function of the distance from the slab (z_d). The working frequency is set at $0.998\omega_c$ where $\omega_c = 3\pi\times10^{14}[1/\mathrm{s}]$. Other parameters are same as those used in Fig. 4(a) of the main text. The peak represents the position of the cylinder slab resonance mode.

We then consider the work needed to be done if one moves the cylinder from $z_d = 0.01d$ to $z_d = 0.5d$ away from the metamaterial slab. The presence of the cylinder-slab resonance provides

an effective potential barrier which prevents the cylinder from being moved away (close) when the working frequency is smaller (larger) than ω_c. In Fig. S4, the work done is plotted as a function of working frequency. The power of the incident light is set at $1\text{mW}/\mu\text{m}^2$ and other parameters are same as in Fig. 4(a) of the main text. The work done is large (compared with the thermal fluctuation energy scale ~ kT at room temperature) when $\omega < \omega_c$ due to the cylinder slab resonance mode and decreases to almost zero when the working frequency reaches ω_c (cloaking). When the working frequency is larger than ω_c, the work done is much smaller because the cylinder slab resonance condition, i.e., $k_o^2 \alpha W_{yy}^{ref} / \varepsilon_o \approx 1$, is not satisfied in this region.

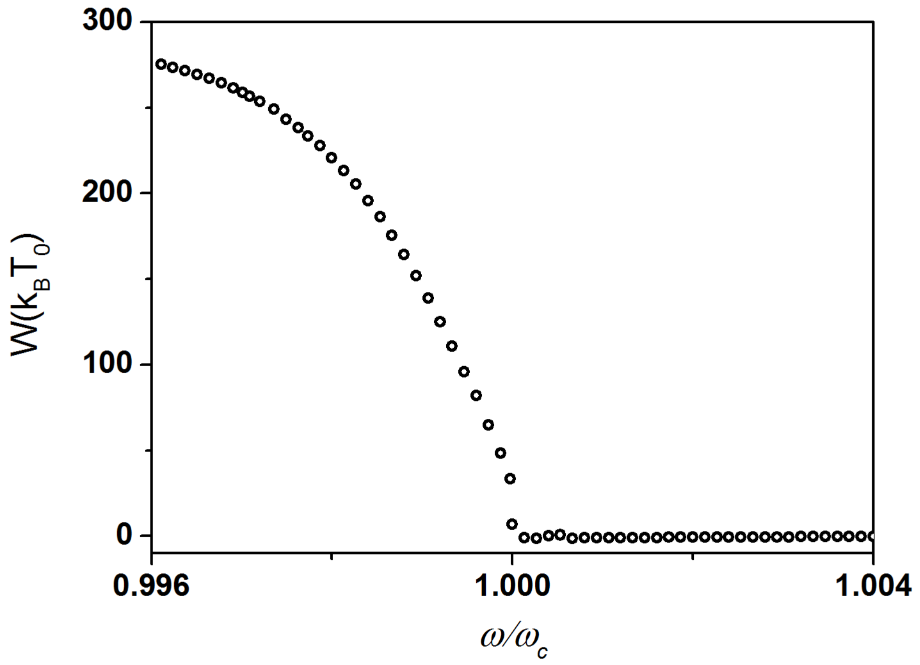

Fig. S4 Work done (in unit of $k_B T_0$, where k_B is the Boltzmann constant and $T_0 = 300\text{K}$ is the room temperature) required to overcome the energy barrier when one moves a cylinder of unit length ($1\mu\text{m}$) from $z_d = 0.01d$ to $z_d = 0.5d$. The direction of incident wave is along the positive z direction and the center frequency and power are set at $\omega_c = 3\pi \times 10^{14}[1/\text{s}]$ and $1\text{mW}/\mu\text{m}^2$, respectively. Other parameters are same as those used in Fig. 4(a).
